# High stability and metabolic capacity of bacterial community promote the rapid reduction of easily decomposing carbon in soil

**DOI:** 10.1038/s42003-021-02907-3

**Published:** 2021-12-08

**Authors:** Ruilin Huang, Thomas W. Crowther, Yueyu Sui, Bo Sun, Yuting Liang

**Affiliations:** 1 State Key Laboratory of Soil and Sustainable Agriculture, Institute of Soil Science, Chinese Academy of Sciences, Nanjing, 210008 China; 2 University of Chinese Academy of Sciences, Beijing, 100049 China; 3 Department of Environmental Systems Science, Institute of Integrative Biology, ETH Zürich, Zürich, Switzerland; 4 Northeast Institute of Geography and Agricultural Ecology, Chinese Academy of Sciences, Harbin, 150040 China

**Keywords:** Climate-change ecology, Microbial ecology

## Abstract

Irreversible climate change alters the decomposition and sequestration of soil carbon (C). However, the stability of C components in soils with different initial organic matter contents and its relationship with the response of major decomposers to climate warming are still unclear. In this study, we translocated Mollisols with a gradient of organic matter (OM) contents (2%–9%) from in situ cold region to five warmer climatic regions to simulate climate change. Soil C in C-rich soils (OM >5%) was more vulnerable to translocation warming than that in C-poor soils (OM ≤ 5%), with a major loss of functional groups like *O*-alkyl, *O*-aryl C and carboxyl C. Variations of microbial β diversity with latitude, temperature and precipitation indicated that C-rich soils contained more resistant bacterial communities and more sensitive fungal communities than C-poor soils, which led to strong C metabolism and high utilization ability of the community in C-rich soils in response to translocation warming. Our results suggest that the higher sensitivity of soils with high organic matter content to climate change is related to the stability and metabolic capacity of major bacterial decomposers, which is important for predicting soil-climate feedback.

## Abstract (web summary)

Huang et al. use a soil translocation experiment to test the effects of higher temperature and initial organic matter content on the stability of soil carbon. Their results demonstrate a link between microbial communities and soil carbon-dependent sensitivity to warming.

## Introduction

The continuous loss of soil organic carbon (SOC) in terrestrial ecosystems has a positive feedback on global warming^1–3^. However, changes in soil C components under warming conditions across soils with different organic matter contents remain unknown, limiting our ability to predict the response of soils to climate change. Some studies have shown that the chemical complexity and changes in the molecular structure of SOC can determine the rates of microbial decomposition and C turnover under increasing temperatures^4,5^. For example, plant-derived polymers, such as lignin, have complex chemical structures that cannot easily decompose within a short time^6,7^, whereas low-molecular-weight compounds, such as proteins, carbohydrates, and fats, are more susceptible to the adverse effects of a warming climate^8^, because lower-molecular-weight C is less expensive to metabolize^9^. The chemical complexity of organic carbon can be inferred from the chemical shifts of functional groups identified by solid-state nuclear magnetic resonance (NMR) spectroscopy, and several studies have shown that the decomposability of C is in the order of *O*-alkyl C > alkyl C > aromatic C^5,10^. Therefore, the rate of SOC decomposition is to some extent related to the proportion of different molecular groups^8,11^. For example, SOC is more likely to be lost when the proportion of *O*-alkyl and di*-O*-alkyl C is high^12^, and SOC is not easily lost when the proportion of alkyl C and aromatic C is high^5^.

The stability of SOC is defined as the tendency of organic C in soils to resist change and/or loss^13^, including resistance and resilience^14^. The stability of SOC to warming is related to its original quantity and lability and can be reduced by changes in microbial physiology, including increased microbial C use efficiency and increased activity of metabolic enzymes^12,15^. Based on the metabolic model^16,17^, it can be inferred that the increase in biochemical reaction rate with temperature will increase the rate of C decomposition by microbes and reduce the total soil C stability. Additionally, the total organic C decomposition rate is closely related to the changes in the microbial community composition caused by climate change. For example, one recent study indicated that the decomposition of litter depended on the change in bacterial and fungal community composition, and faster bacterial community turnover had a more significant impact on litter decomposition rates than fungi^18^. However, previous studies revealed that the effects of the community composition on soil C stability were mainly reflected in the overall C decomposition or turnover rate^12,18,19^, and it remains unclear which specific C components are more vulnerable to changes in the community composition.

In a single ecosystem with relatively little disturbance, species sorting may lead to a single, “functionally optimal” community, composed of taxa with a series of traits and population densities that are well suited to the exploitation of available resources^20^. However, this “functionally optimal” community is susceptible to environmental changes (e.g., climate warming) and may ultimately affect the rate of decomposition. Studies have demonstrated that environmental heterogeneity is significantly and positively correlated with changes in the microbial community composition (i.e., β diversity)^20^ and that their interactions alter the decomposition rates^9^. This finding may be due to the fact that changes in the environment in which microorganisms live can cause changes in microbial interactions, such as resource competition and metabolic cross-feeding^21^, which may reduce (e.g., through competitive exclusion) the functional redundancy of the decomposer community. This finding implies that differences in resource availability may largely affect the reassembly and catabolic functions of the microbial community. Moreover, due to the divergent sensitivity to environmental changes, different microbial communities (e.g., bacteria and fungi) may show different responses to climate change even under the same resource state^22^. However, the responses of different important decomposers to climate change under different resource states (e.g., organic carbon content) remains elusive.

**Fig. 1:**
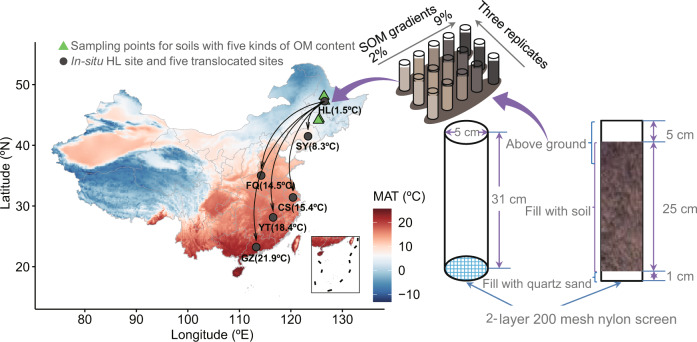
Schematic diagram of the experimental design. The green-filled triangles are the sampling points for soil with five different OM contents. The dark gray-filled circles are the in situ HL site and the five translocated sites. Fifteen samples (five SOM contents × three replicates) were collected at each site. The black solid lines with arrows show the direction of the soil translocation.

Field-based translocation experiments provide an opportunity to elucidate the effects of simultaneous increases in temperature and precipitation on the relationship between changes in microbial communities and soil C stability, which can deepen our understanding of the ecological consequences of integrated climate change in the field^23,24^. Here, we manipulated soil communities across a broad climatic gradient through the southward translocation of soils (Mollisols based on the FAO classification^25^) with different contents of original soil organic matter (SOM; in the range of 2%~9% SOM kg^−1^ soil^−1^) from the middle temperate zone to the warm temperate zone and the subtropical zone along the temperature gradient (Fig. 1). Warming due to translocation was used as a proxy for climate change integrating changes in both precipitation and temperature. One year after soil translocation, the changes in SOC functional groups were measured between the in situ and translocated climatic regimes. Here, we propose the following hypotheses: (1) a high stability of microbial communities is expected to lead to an increase in C loss in the presence of increased metabolism due to translocation-related warming, because stable microbial communities can maximize the maintenance of the original “functionally optimal” community structure^26,27^; and (2) compared with soil with low SOM, soil with high SOM has a stronger buffering capacity to the changes in nutrient availability and soil pH caused by climate change^28,29^. Therefore, important decomposers (e.g., bacteria and fungi) in soil with high SOM exhibit higher stability under translocating warming, which leads to a large level of decomposition of easily decomposable C. Our results indicated that compared with that found in C-poor soil, the decomposition of organic C in soils with high SOM was faster and dominated by easily decomposable functional groups. This difference was related to the higher stability and metabolic capacity of the bacterial community.

## Results

### Responses of SOC and C molecular groups to translocation warming

**Fig. 2:**
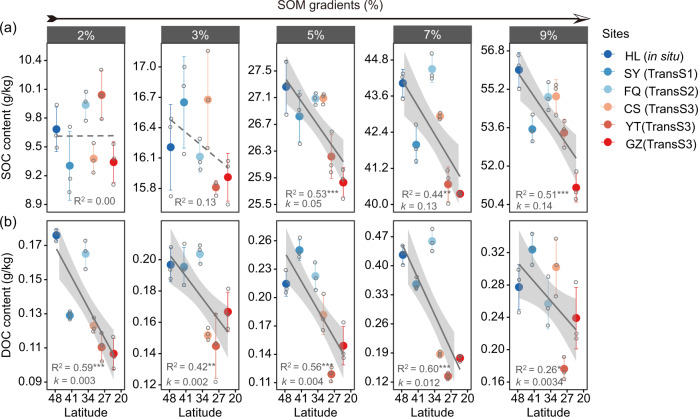
Responses of soil C to climate change. Linear fitting analysis for SOC (**a**) and DOC (**b**) and latitude. The fitting curve type was determined by comparing the Akaike information criterion (AIC) value. The solid and dashed lines represent significant (*p* < 0.05) and nonsignificant (*p* > 0.05) models, respectively. The different-colored circles represent different sampling sites from high to low latitudes. *R*^2^ and *k* represent the amount of interpretation and the fitting slope of the model, respectively. The error bar is the standard deviation (*n* = 3). Significance is presented by ^*^p < 0.05, ^**^p < 0.01, and ^***^p < 0.001.

To investigate the response of organic C to climate change in the same soil types (Mollisols) with different original organic matter contents, we conducted a climate gradient translocation experiment with gradients of organic matter. The established gradients include bulk soil and entire microbial communities. Specifically, our experimental design involved the collection of soils with five organic matter contents (2%, 3%, 5%, 7%, and 9% SOM kg^−1^ soil^−1^) from three adjacent sites with the same climate type and the subsequent translocation of these soils to five warmer regions with different climate types (Fig. 1). All SOM gradient soils were included in each site. A linear fitting analysis of SOC and dissolved organic carbon (DOC) versus latitude for all 90 samples across the five organic matter levels was performed to assess the response of total SOC and specific different C components (e.g., labile carbon) to translocation warming. The results showed that 1 year after soil translocation, the changes in the organic C content were clearly closely related to the proportion of original SOM in soils (Table S4). Considerable SOC loss only occurred in soils with a SOM content in the range of 5–9% (approximately 0.4% loss per 1 °C) (Fig. 2a). DOC also lost more in C-rich soils than in C-poor soils (approximately twice as fast in C-rich soils as in C-poor soil, Fig. 2b).

**Fig. 3:**
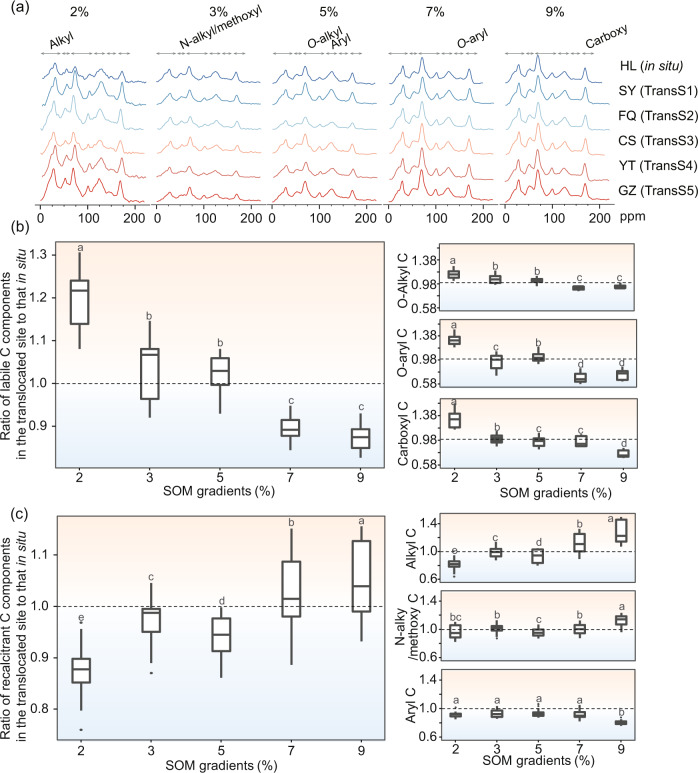
Response of different functional groups of SOC to translocation warming. In the boxplot, the middle line of the box is the median of the data. The upper and lower limits of the box are the upper and lower quartiles of the data, respectively. ^13^C solid-state NMR spectra of SOC with different molecular structures (**a**). Ratios of the abundance of labile C (**b**) and recalcitrant C (**c**) components in the five translocation sites to the abundance of the corresponding C components in the in situ HL site. Compared with in situ site, a ratio greater than 1 indicates that translocation-related warming increases the stability of carbon, whereas a ratio less than 1 shows that translocation-related warming reduces the stability of carbon. The differences in C stability among soils with different organic matter contents were statistically determined using TukeyHSD method. Different lowercase letters indicate significant differences, and the same lowercase letters indicate nonsignificant differences.

In addition, we evaluated the response of specific C components with different functional groups to translocation warming based on solid-state ^13^C NMR spectroscopy (Fig. 3a and Supplementary Data 1). The results showed that at the original climatic site (HL), SOC was dominated by recalcitrant C (e.g., alkyl and *N*-alkyl/methoxy C) in C-poor soils and by labile C (e.g., *O*-alkyl, *O*-aryl C, and carboxyl C) in C-rich soils (Fig. S1 in the Supplementary information). Different molecular functional groups exhibited asymmetric responses to translocation warming (Fig. 3b, c). Compared with the coldest HL site, translocation warming reduced (~11.48% reduction) labile C and increased (~3.79% increase) recalcitrant C in C-rich soils. In contrast, translocation warming markedly reduced (~7.16% reduction) recalcitrant C and increased (~8.43% increase) labile C in C-poor soils.

### Changes in the bacterial and fungal communities in response to translocation warming

**Fig. 4:**
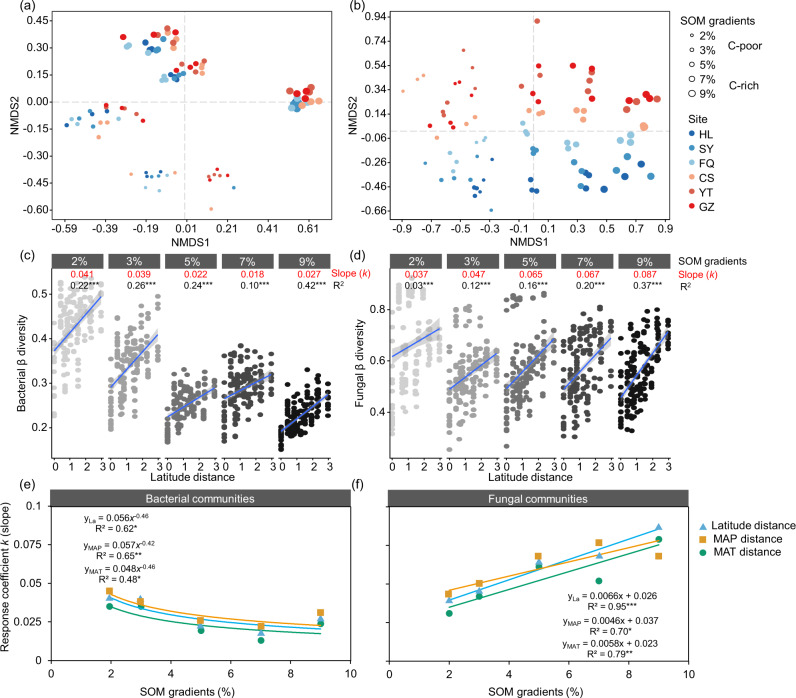
Effects of SOM gradients and climate change on the microbial community composition. Nonmetric multidimensional scaling (NMDS) analysis of bacterial (**a**) and fungal communities (**b**). The size and color of the circle represent the SOM content and the different sampling sites from north to south, respectively. Linear fitting relationships of the latitude distance with the bacterial β-diversity (**c**) and the fungal β-diversity (**d**). The variable *k* (slope) is the response coefficient of β to the distance of latitude (climate change). In this study, the response coefficient represents the rate of change in the community composition. *R*^2^ is the amount of interpretation of the model. Relationships of the content of SOM with the climatic response coefficients of the bacterial (**e**) and fungal (**f**) β-diversity. These climatic response coefficients include the response coefficients (*k*) of the β-diversity to changes in latitude, MAT, and MAP. The circles with different colors represent the climatic response coefficients of bacteria and fungi to different factors, including latitude (sky blue), MAP (orange), and MAT (bluish green).

To test hypothesis 2 regarding whether the community structure of bacteria and fungi was more stable in soils with a high organic matter level than in soils with a low organic matter level under translocation warming, we analyzed the responses of bacterial and fungal communities to translocation warming using various statistical methods (see “Methods”). Consistent with our expectation, the results showed that the SOM content was the dominant factor affecting bacterial and fungal communities, followed by climatic factors (Fig. 4a, b and Table 1). Nonetheless, the relationship between the stability of the bacterial and fungal communities under translocation warming and the original SOM content was not the same. Specifically, bacteria were more sensitive than fungi to the SOM content, and fungi were more susceptible than bacteria to climate change (*p* < 0.001, Table 1). Furthermore, the linear fitting analysis showed that the β-diversity of bacterial and fungal communities was significantly positively correlated with latitude changes (*p* < 0.001, Fig. 4c, d) and climatic factors (Fig. S2). The response coefficient (*k*, representing the variation rate of the community composition) of the bacterial β-diversity to changes in latitude, mean annual precipitation (MAP), and mean annual temperatures (MAT) was negatively correlated with the SOM content (Fig. 4e, average of 0.041–0.018), whereas that of fungal β-diversity was positively correlated with the SOM content (Fig. 4f, average of 0.036–0.078). These results indicate that bacterial communities are more stable and that fungal communities are more sensitive to translocation warming in C-rich soil.

**Table 1:** Significance tests of the effects of SOM content and translocation warming on bacterial and fungal community structure with three different statistical approaches.

	Microbes	Adonis^a^		ANOSIM^b^		MRPP^c^	
		*F*	*p*^d^	*R*	*p*^d^	*δ*	*p*^d^
SOM	Bacteria	26.90	<0.001	0.902	<0.001	0.359	<0.001
	Fungi	11.98	<0.001	0.64	<0.001	0.18	<0.001
Translocation warming	Bacteria	4.85	<0.001	0.11	<0.001	0.039	<0.001
	Fungi	8.20	<0.001	0.29	<0.001	0.075	<0.001

All three tests are nonparametric multivariate analysis based on dissimilarities among samples.

^a^Permutational multivariate analysis of variance using distance matrices. Significance tests were carried out using *F*-tests based on sequential sums of squares from permutations of the raw data.

^b^Analysis of similarities. Statistic R is based on the difference of mean ranks between groups and within groups. The significance of observed *R* is assessed by permuting the grouping vector to obtain the empirical distribution of *R* under the null model.

^c^Multi-response permutation procedure. Statistic *δ* is the overall weighted mean of within-group means of the pairwise dissimilarities among sampling units. The significance test is the fraction of permuted *δ* that is less than the observed *δ*.

^d^*p*-Value of corresponding significance test.

A network analysis was performed to understand the potential interaction patterns of bacterial and fungal communities in C-poor and C-rich soils (Fig. S3a, b). The microbial network structure in response to translocation warming changed slightly (average change in the total number of edges was approximately 5%) in C-rich soils but was significantly altered (average change in the total number of edges was approximately 50%) in C-poor soils (Table S1). The negative associations between the bacterial fungal communities increased by 2.9- to 3.9-fold (Fig. S4). The network topology parameters showed that the network complexity and connectivity of the community in C-rich soils were consistently higher than those in C-poor soils (Table S1).

### Soil microbial metabolism of diverse C sources

**Fig. 5:**
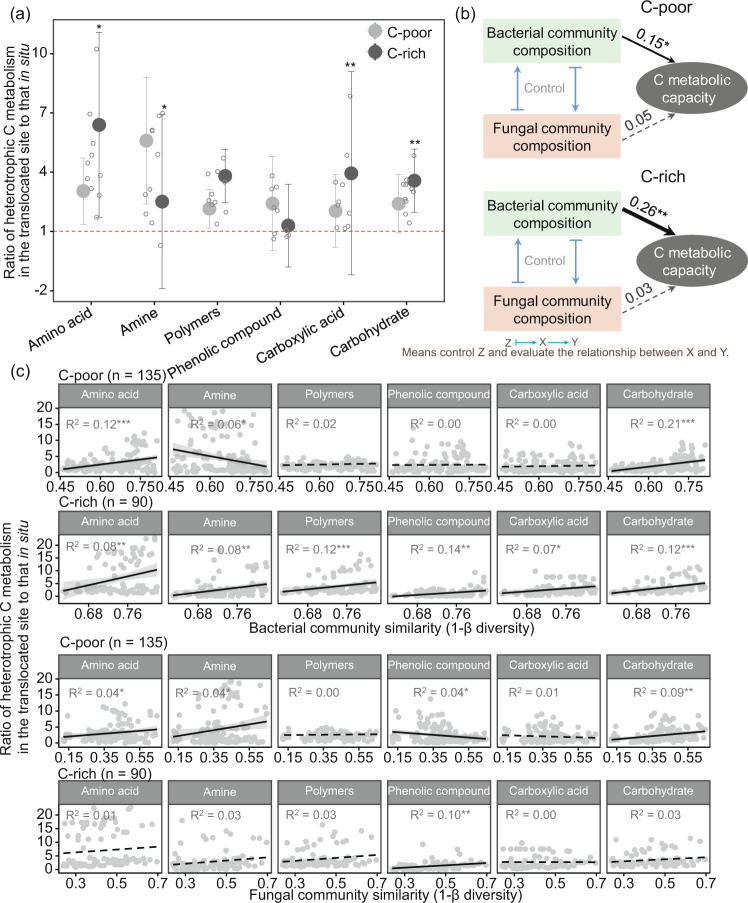
Relationship between C metabolic capacity and microbial communities. C metabolic capacity (i.e., standard OD values) of different types of C sources in C-poor and C-rich soils (**a**). Compared with the in situ (HL) site, a ratio greater than 1 indicates that translocation-related warming increases the C metabolism of microorganisms, whereas a ratio less than 1 shows that translocation-related warming reduces the C metabolism of microorganisms. Significance was assessed by a paired *t*-test of the mean C metabolic capacity of five translocated sites in C-poor vs. C-rich soils. The error bar represents the standard deviation (*n* = 5). Partial Mantel analysis between bacterial and fungal community composition and C metabolic capacity in C-poor and C-rich soils (**b**). The black solid lines with arrows represent significant correlations, whereas the dashed gray lines represent nonsignificant correlations. The numbers adjacent to the arrows are correlation coefficients. Fitting analysis between the bacterial and fungal community similarity and C metabolism changes (**c**). Both community similarity and C metabolism changes were obtained and compared with those in the in situ HL site. Significance is presented by ^*^p < 0.05, ^**^p < 0.01, and ^***^p < 0.001.

Based on BIOLOG 96-well Eco-Microplates, all 90 soil samples were incubated with 31 C sources to profile the soil microbial C metabolic capacity (Table S2). Compared with the results obtained with the coldest HL site, translocation warming substantially increased the microbial C decomposition capacity (Fig. S5). The promotion effect of translocation warming on the C metabolic capacity of amino acids, polymers, and carbohydrates was significantly higher in C-rich soils than in C-poor soils (*p* < 0.05, Fig. 5a). A partial Mantel analysis showed that bacteria were more related to the community heterotrophic decomposition capacity than fungi (Fig. 5b). When fungal communities were controlled, changes in the bacterial community composition significantly affected the community C metabolic capacity (*p* < 0.05). Furthermore, a fitting analysis between the community β-diversity of bacteria and fungi and the metabolism of six categories of C sources showed a significant positive correlation between the community similarity and the heterotrophic decomposition capacity, particularly for bacterial communities in C-rich soil (Fig. 5c).

### Relationships of biotic and abiotic factors to soil labile and recalcitrant C

**Fig. 6:**
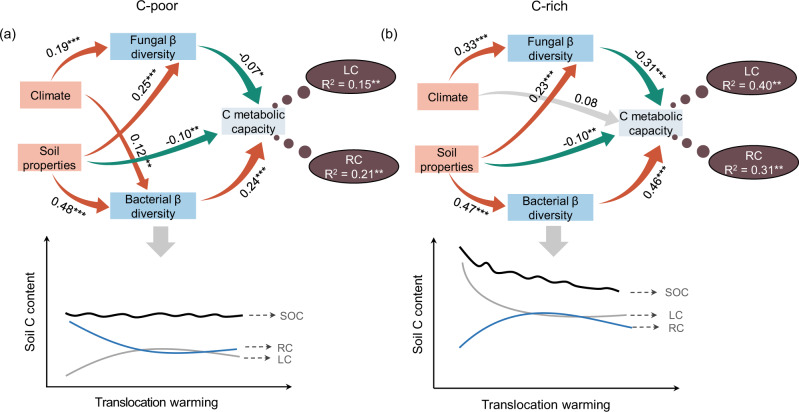
Effects of biotic and abiotic factors on soil organic carbon. Structural equation models (SEMs) of soil properties, climatic conditions, bacterial and fungal community β-diversity and C metabolic profiles in C-poor (**a**) and C-rich soils (**b**). SEM of C-poor soils, *χ*^2^/d*f* = 0.37, *p* = 0.54; AIC = 28; GFI = 0.99, AGFI = 0.99; RMSEA = 0.00, *p* = 0.91; Bootstrap *p* = 0.52. SEM in C-rich soils, *χ*^2^/d*f* = 0.63, *p* = 0.43; AIC = 29; GFI = 0.99, AGFI = 0.99; RMSEA = 0.00, *p* = 0.70; Bootstrap *p* = 0.39. The red, green, and gray lines with arrows represent significant positive, significant negative, and nonsignificant correlations, respectively. The numbers adjacent to the arrows are correlation coefficients. The brown dashed lines represent random forest analyses of the importance of the C metabolic capacity for labile C (LC) and recalcitrant C (RC) in C-poor and C-rich soils. *R*^2^ is the amount of interpretation of the model. Significance is presented by ^*^p < 0.05, ^**^p < 0.01, and ^***^p < 0.001.

To test hypothesis 1, structural equation models (SEMs) were fitted to determine the direct and indirect effects of soil properties (pH, moisture, ammonia, and nitrate nitrogen), climatic conditions (MAT and MAP), and the bacterial and fungal community structure on soil labile C and recalcitrant C in C-poor and C-rich soils (Fig. 6a, b). The results showed that the changes in soil properties and climatic conditions caused by soil translocation could affect the soil C metabolic capacity by affecting the β-diversity of bacterial and fungal communities. Compared with the results obtained with C-poor soil, translocation warming had less impact on the bacterial β-diversity but more impact on the fungal β-diversity in C-rich soil. The bacterial β-diversity was a major biotic factor affecting the community C metabolic capacity, explaining 24% and 46% of the variations in C metabolism in C-poor and C-rich soils, respectively (Fig. S6). A random forest model analysis showed that the increase in C metabolism in C-poor soil mainly enhanced the decomposition of recalcitrant C (alkyl, *N*-alkyl/methoxy, and aryl C) by microbes, and the increase in C metabolism in C-rich soil mainly enhanced the decomposition of labile C (*O*-alkyl, *O*-aryl C, and carboxyl C) by microbes (Fig. 6). These results indicated that a large amount of C loss in C-rich soils within a short term was mainly caused by the highly stable bacterial communities, which decomposed more labile C.

## Discussion

Larger soil C stocks are generally reported to be more susceptible to climate warming. Specifically, warming can cause considerable C losses in soil with C stocks that exceed 7 kg C m^−2^ (equivalent to 26.42 g C kg^−1^ soil^−1^)^2^. Similarly, our results showed that organic C in C-rich soils (>5% SOM, equivalently 28 g C kg^−1^ soil^−1^) was more vulnerable to translocation warming than that in C-poor soils (Fig. 2). Soil C is mainly composed of labile C (*O*-alkyl, *O*-aryl, and carboxyl C) in C-rich soils and recalcitrant C (alkyl, *N*-alkyl/methoxy, and aryl C) in C-poor soils (Fig. S1). Because most microbes can use labile C as an energy source^12,30^ and metabolize labile C faster^19^, soil C in C-rich soils, particularly labile C, is more susceptible to translocating warming. In addition, regular weeding was performed in our experiments to reduce the input of plant-derived C. If there is C input from plants, the result may be different, because a large amount of plant-derived C input may offset the warming-induced loss of C^2,31^.

In our study, the difference in the total C content could not be detected in soils with a low organic matter content; however, analysis at a finer molecular level revealed that the responses of different C substrates to translocation-related warming varied considerably. In C-poor soils, the stability of recalcitrant C decreased significantly, whereas that of labile C increased significantly (Fig. 3b, c). This difference may be due to the dominance of oligotrophic *k*-strategic microbes (e.g., *Acidobacteria* and most fungi, see Fig. S7), which prefer to use recalcitrant C^32^. Based on the continuum model of SOM^33^ and the trade-off balance of molecular functional groups^11^, the microbial degradation of recalcitrant C may increase the accumulation of *O*-alkyl, *O*-aryl C, and carboxyl C molecules because large-molecular-size OM will continue to form small-molecular-size organic compounds during the degradation process. Hence, in C-poor soils, translocation warming may increase the stability of easily degradable groups but decrease that of groups with resistance to degradation.

Our results indicated a significantly positive relationship between the similarity of the microbial communities and C metabolic ability in C-rich soils (Fig. 5c). This finding implies that the composition of the initial microbial community has a greater influence on the heterotrophic decomposition capacity under translocation warming^18,26^. This effect occurs because microbial communities with high stability (mainly referring to the ability of the community to resist translocation warming) may play a more critical role in retaining the original “functional optimal” community and maintaining ecosystem function than communities with low stability^20,34^. Moreover, the stability of the microbial community structure may affect the stability of potential network interactions among microbes.

Here, we found that the microbial network connectivity and stability in C-rich soils were higher than those in C-poor soils, and these features are closely related to the multifunctionality of ecosystems^35–37^ and C utilization and allocation^38^. This effect may arise because a high proportion of unstable C substrate in C-rich soils favors the growth and reproduction of microbes with low substrate affinity and high growth rates, such as some members of *Proteobacteria*, *Actinobacteria*, and *Bacteroidetes*^30,32,39^. In C-rich soils, labile C molecules such as *O*-alkyl, *O*-aryl, and carboxyl C molecules are expected to be rapidly utilized by these microbes, resulting in total C loss.

Consistent with previous studies^22,40^, we found that the bacterial community structures under translocation warming were more similar to those in situ than the structures of the fungal community (Fig. S8), which indicates a high resistance to climate change. The high abundance, widespread dispersal, and potential for rapid growth rates all help bacterial communities adapt to new environmental conditions and maintain the composition of the original community to the maximum extent^26^. In parallel, a higher diversity of bacteria that can be selected for by environmental filtering and a more rapidly evolved response of bacteria to short-term climate change also contribute to a more rapid response of the bacterial composition to climate change^41,42^. Although some eukaryotic fungi may exhibit high individual resistance with high physiological tolerance and metabolic flexibility^43^, the resistance of the community structure is reflected in the consistency of the resistance of a community’s members to disturbances.

The analysis of the variation coefficients of the microbial β-diversity with changes in latitude, temperature and precipitation showed that C-rich soils contained more resistant bacterial communities but more sensitive fungal communities than C-poor soils. Bacteria are more sensitive to changes in soil properties, such as pH and nutrient availability^40^. Here, redundancy analysis (RDA) showed that soil properties (such as SOC, TN, and soil pH) contributed to twice as much of the variation in the bacterial community structure as in the fungal community (Fig. S9). A high SOM content can buffer the disturbance of soil resources (e.g., nutrient availability, moisture, and pH) caused by climate change^28,29^. The coefficient of variation and principal component analysis showed that changes in soil resources, particularly nitrate nitrogen (NO_3_^−1^-N), were notably lower in C-rich soils under translocation warming than in C-poor soils (Fig. S10). In addition, the stability of the fungal community structure decreased with increases in the OM content (Fig. 4d, f), which may be due to the enhancement of fungal survival pressure transmitted by bacteria with increasing nutrient availability^38,44^. Studies have reported that the competitive advantage of bacteria and fungi varies depending on the nutritional status available in their environment^45,46^. In general, slow-growing microbes tend to have a high competitive advantage in nutrient-poor environments, whereas fast-growing microbes dominate in nutrient-rich environments^47,48^. According to previous studies, bacteria grow more rapidly with high nutrient requirements than fungi^44,49^. Therefore, the bacterial communities in C-rich soils may transmit the effects of higher pressure (such as nutrient competition) on fungal communities, thus reducing the stability of fungal community structure.

In addition, although our results suggest that stable bacterial community structures contribute to C decomposition under translocation warming, the dynamic change in this relationship needs to be studied and verified over a longer time period. As bacterial and fungal communities persistently adapt to warming, how will the relationship between changes in their community composition and C decomposition change? Second, because the C-degrading functional genes of microorganisms determine their C-degrading potential^15^, it is necessary to evaluate the response of bacterial and fungal functional genes to climate warming and their relationship with SOC stability. In addition, it should be noted that in the case of translocation warming, higher microbial life turnover is likely to partially offset the loss of C by increasing the microbial necromass (i.e., microbial residuals)^50–52^. The balance between C input from microbial residues and the decomposition of old C may be one of the factors affecting the stability of SOC. Therefore, future research should focus on quantitatively evaluating the effects of microbial physiology and necromass on SOC stability in C-poor and C-rich soils under global warming.

## Methods

### Site characteristics and experimental design

In this study, agricultural soils with five SOM contents were collected in 2015 from the following three different locations with the same climate type (the moderate temperate continental climate) in Northeast China (Table S3 and Fig. 1): Bei'an (BA), Hailun (HL), and Dehui (DH). Their MAT and MAP range from 1.0 to 4.4 and 520 to 550, respectively. After collection, the samples were transported to the Hailun Agricultural Ecological Experimental Station (HL), where the samples were packed into the same PVC tubes. Moving the soil from these three initial sampling points to the HL may have had some influence on the microbes, but compared with longer-distance soil translocation across different climatic zones, the HL site can be regarded as an in situ site that reflects the original climatic conditions. The SOM contents were 2%, 3%, 5%, 7%, and 9% (equivalent to 10, 18, 28, 36, and 56 g C kg^−1^ soil^−1^, respectively), and all the soils were classified as Mollisols according to the FAO classification. Here, we designed a unique latitudinal soil translocation experiment to investigate the relationship between the bacterial and fungal community stability and the responses of soil C molecular structure to climate warming. The detailed protocol for the experiment was the following: (1) Forty kilograms of topsoil (0–25 cm) was collected for each SOM. The latitude and longitude of the sampling sites and soil geochemical characteristics are shown in Tables S3 and S4. Detailed data can be found in Supplementary Data 1. (2) The soil was homogenized using a 2 mm sieve and filled with sterilized PVC tubes. The PVC tube was 5 cm in diameter at the bottom and 31 cm in height. Each tube was filled with a 25 cm-high soil column, which corresponded to approximately 1 kg of soil. The bottom of the pipe was filled with 1 cm quartz sand, and a 5 cm space was left at the top. (3) From October to November 2015, 90 PVC pipes containing soil (5 SOM gradients × 3 replicates × 6 climatic conditions) were transported to six ecological research stations with different geoclimatic conditions and SOM contents, and 15 PVC pipes were placed in each station. Once the experiment was set up, the weeds growing in each PVC pipe were manually removed every 2–3 weeks to avoid the impact of plants.

The six ecological research stations were the Hailun Agricultural Ecological Experimental Station (HL, N 47°27′, E 126°55′) in Heilongjiang Province, Shenyang Agriculture Ecological Experimental Station (SY, N 41°49′, E 123°33′) in Liaoning Province, Fengqiu Agricultural Ecological Experimental Station (FQ, N 35°03′, E 114°23′) in Henan Province, Changshu Agricultural Ecological Experimental Station (CS, N 31°41′, E 120°41′) in Jiangsu Province, Yingtan Red Soil Ecological Experiment Station (YT, N 28°12′, E 116°55′) in Jiangxi Province and Guangzhou National Agricultural Science and Technology Park (GZ, N 23°23′, E 113°27′) in Guangdong Province. The MAT and MAP at the six ecological research stations ranged from 1.5 to 21.9 °C and from 550 to 1750 mm from north to south, respectively. Details of their climatic conditions (e.g., climatic types) are shown in Table S5. All tubes were removed from each station after 1 year.

The soil samples were stored on dry ice and rapidly transported back to the laboratory. The soil pH was measured by the potentiometric method. Nitrate (NO_3_^−^-N) and ammonium nitrogen (NH_4_^+^-N) were measured by the Kjeldahl method. DOC was measured using a total organic carbon analyzer (Shimadzu Corporation, Kyoto, Japan). SOC was determined by wet digestion using the potassium dichromate method^53^. Microbial biomass C (MBC) was measured by the chloroform fumigation-incubation method^54^. All geochemical attributes are shown in Table S4.

### Solid-state ^13^C NMR analysis of soil C molecular groups

Solid-state ^13^C NMR spectroscopy analysis was performed to determine the molecular structure of SOC. A Bruker-Avance-iii-300 spectrometer was used at a frequency of 75 MHz (300 MHz 1H). Before the examination, the soil samples were pretreated with hydrofluoric acid to eliminate the interference of Fe^3+^ and Mn^2+^ ions in the soil. Specifically, 5 g of air-dried soil was weighed in a 100 ml centrifuge tube with 50 ml of hydrofluoric acid solution (10% v/v) and shaken for 1 h. The supernatant was then removed by centrifugation at 3000 rpm for 10 min. The residues were washed eight times with a hydrofluoric acid solution (10%) with ultrasonication. The oscillation program consisted of the following: four × 1 h, three × 12 h, and one × 24 h. The soil samples were washed with distilled water four times to remove the residual hydrofluoric acid. The above-mentioned treated soil samples were dried in an oven at 40 °C, ground and passed through a 60-mesh sieve for NMR measurements.

The soil samples were then subjected to solid-state magic-angle rotation-NMR measurements (AVANCE II 300 MH) using a 7 mm CPMAS probe with an observed frequency of 100.5 MHz, an MAS rotation frequency of 5000 Hz, a contact time of 2 s, and a cycle delay time of 2.5 s. The external standard material for the chemical shift was hexamethyl benzene (HMB, methyl 17.33 mg kg^−1^). The spectra were quantified by subdividing them into the following chemical shift regions^55^: 0–45 ppm (alkyl), 45–60 ppm (*N*-alkyl and methoxyl), 60–110 ppm (*O*-alkyl), 110–140 ppm (aryl), 140–160 ppm (*O*-aryl), 160–185 ppm (carboxy), and 185–230 ppm (carbonyl) (Fig. 3a). We classified *O*-alkyl, *O*-aryl, and carboxy C as labile C and alkyl, *N*-alkyl/methoxyl, and aryl C were classified as recalcitrant C.

### Soil microbial C metabolic profiles

The soil microbial C metabolic capacities were measured with BIOLOG 96-well Eco-Microplates (Biolog Inc., USA) using 31 different C sources and three replicates in each microplate. These C sources included carbohydrates, carboxylic acids, polymers, amino acids, amines, and phenolic acids (Table S2). Carbohydrates, amino acids, and carboxylic acids are generally considered labile C sources, amines and phenolic acid compounds are relatively resistant C sources, and polymers are recalcitrant C. The diverse nature of these C sources allowed us to identify differences in the capacity of microbes to degrade different C sources^56^. Soil microbes were extracted as follows: (1) Five grams of soil (dry weight equivalent) was incubated at 25 °C for 24 h, and 45 ml of sterile 0.85% (w/v) sodium chloride solution was added^57^. (2) At room temperature (25 °C), the mixture was shaken at 200 rpm for 30 min and allowed to stand for 15 min. (3) Subsequently, 0.1 ml of the supernatant was collected and diluted to 100 ml with sterile sodium chloride solution. (4) Soil suspensions were dispensed into each of the 93 wells (150 μl per well), and the plates were then incubated at 25 °C in the dark for 14 days. The optical density (OD, reflecting C utilization) of each well was read at 590 nm (color development) every 12 h. The normalized OD of different C sources was calculated as the OD of the well that contained the C source minus the OD of the well that contained sterile sodium chloride solution (control well). The normalized OD at a single time point (228 h) was used for the posterior analysis when it reached the asymptote.

### DNA extraction, PCR amplification, and sequencing

DNA was extracted from all 90 soil samples. Briefly, well-mixed soil samples (0.6 g) were analyzed using the Power Soil DNA Isolation Kit (MoBio Laboratories, Inc., Carlsbad, CA, USA) following the manufacturer’s instructions. The quality of the DNA extracts was determined by spectrophotometry (OD-1000^+^, OneDrop Technologies, China). The DNA extracts were considered of sufficient quality if the ratio of OD_260_ to OD_280_ (optical density, OD) and the ratio of OD_260_ to OD_230_ were approximately 1.8. All eligible DNA samples were stored at −80 °C.

Taxonomic profiling of the soil bacterial and fungal communities was performed using an Illumina^®^ HiSeq Benchtop Sequencer. PCR amplification was performed using an ABI GeneAmp^®^ 9700 (ABI, Foster City, CA, USA) with a 20 μl reaction system containing 4 μl of 5× FastPfu Buffer, 0.8 μl of each primer (5 μM), 2 μl of 2.5 mM dNTPs, 2 μl of template DNA, and 0.4 μl of FastPfu Polymerase. For bacterial analysis, the forward the primer 515F (GTGCCAGCMGCCGCGG) and the reverse primer 907R (CCGTCAATTCMTTTRAGTTT) were used to amplify the bacteria-specific V4-V5 hypervariable region of the 16S rRNA gene^58^. For fungal analysis, the internal transcribed spacer 1 (ITS1) region of the ribosomal RNA gene was amplified with primers ITS1-1737F (GGAAGTAAAAGTCGTAACAAGG) and ITS2-2043R (GCTGCGTTCTTCATCGATGC)^59^. The PCR protocol for bacteria consisted of an initial predenaturation step of 95 °C for 2 min, 35 cycles of 20 s at 94 °C, 40 s at 55 °C and 1 min at 72 °C, and a final 10 min extension at 72 °C. The PCR protocol for fungi consisted of an initial predenaturation step of 95 °C for 3 min, 35 cycles of 30 s at 95 °C, 30 s at 59.3 °C, and 45 s at 72 °C and a final 10 min extension at 72 °C.

Each sample was independently amplified three times. Following amplification, 2 μl of each of the PCR products was checked by agarose gel (2.0%) electrophoresis, and all the PCR products from the same sample were then pooled together. The pooled mixture was purified using the Agencourt AMPure XP Kit (Beckman Coulter, CA, USA). The purified products were indexed in the 16S and ITS libraries. The quality of these libraries was assessed using Qubit@2.0 Fluorometer (Thermo Scientific) and Agilent Bioanalyzer 2100 systems. These pooled libraries (16S and ITS) were subsequently sequenced with an Illumina HiSeq 2500 Sequencer to generate 2 × 250 bp paired-end reads at the Center for Genetic & Genomic Analysis, Genesky Biotechnologies Inc., Shanghai, China.

The raw reads were quality filtered and merged as follows: (1) TrimGalore was used for truncation of the raw reads at any site with an average quality score <20, removal of reads contaminated by the adapter and further removal of reads with less than 100 bp; (2) the paired-end reads were merged to tags by Fast Length Adjustment of Short reads (FLASH, v1.2.11); (3) the removal of reads with ambiguous base (N base) and reads with more than 6 bp of homopolymer was performed with Mothur; (4) reads with low complexity were then removed to obtain clean reads for further bioinformatics analysis. The remaining unique reads were chimera checked by comparison with the gold.fa database (http://drive5.com/uchime/gold.fa) and clustered into operational taxonomic units (OTUs) using QIIME2 with a 97% similarity cutoff. The Ribosomal Database Project classifier performed the taxonomic assignment of OTUs with a minimal 70% confidence score^60^. For 16S data, taxonomic assignment was performed using the SILVA database (version 138, https://www.arb-silva.de/)^61^; for the ITS, the UNITE database was used (version 8.2, https://unite.ut.ee/)^62^.

To obtain the relationship between the number of detected species and the sequencing depth, we performed a linear fitting analysis between the number of OTUs and the number of reads (Fig. S11). The results showed that the number of species (OTUs) detected in the samples tended to level off once the number of bacterial and fungal reads was higher than 42,254 and 54,932, respectively (Fig. S11a, b). In this study, from all 90 samples, 14,414 OTUs were acquired for the bacterial community, and their read numbers ranged from 42,254 to 150,465; for the fungal community, a total of 9811 OTUs were obtained, and their read numbers ranged from 54,932 to 492,981. In the subsequent bioinformatics analysis, to minimize the impact of changes in the reading count among different samples, we rarefied all the samples based on the smallest read numbers (42,254 bacterial reads and 54,932 fungal reads per sample).

### Microbial co-occurrence network construction

CoNet was used to generate interaction networks to determine the effects of climate change on the connections of bacteria and fungi in C-poor and C-rich soils. Zero-rich data were filtered before network construction. Briefly, the construction of a network graph was divided into four steps: basic configuration, permutation, bootstrapping, and restoration of the network from random files. Pairwise associations among OTUs were calculated using the Pearson, Spearman, Bray-Curtis, and Kullback-Leibler methods simultaneously. The initial top and bottom edge numbers were set to 1000. An edge- and measure-specific *p*-value was obtained as the area under the bootstrap distribution limited by the mean of the permutation distribution^63^. The edges were retained when supported by at least two correlation methods. The edges were discarded when the 95% confidence interval defined limits by the bootstrap distribution or the adjusted *p*-values were higher than 0.05. A final network was restored from the permutation and bootstrap files^64^. Graphs of the interaction network were built and visualized using Cytoscape (version 3.8.1). Each node in the network represents a bacteria/fungus, and the edges represent the correlation among different nodes.

### Statistics and reproducibility

#### Analysis of changes in the SOC content and microbial C metabolism capacity

The relationships of SOC and DOC with latitude were evaluated using a fitting analysis. The fitting model corresponds to the minimum Akaike information criterion (AIC) value as the final model (16 samples under each OM gradient). In soils with the same OM, the relative abundance of C molecules in the translocated sites was divided by the relative abundance of C molecules in the in situ HL site to evaluate the stability of different C components (i.e., alkyl, *O*-alkyl, *N*-alkyl/methoxyl, aryl, *O*-aryl, carboxy C) under elevated temperatures. A total of 45 ratios were obtained for each OM content. A ratio greater than 1 represents increased stability, and a ratio less than 1 represents decreased stability. Similarly, in C-poor (OM ≤ 5%) and C-rich (OM > 5%) soils, changes in the C metabolic capacity of microbes under elevated temperatures were characterized using the ratio of the OD of microbes measured in the translocated soils to the OD of microbes in the in situ HL soil. A ratio greater than 1 indicates that translocation warming increases the C metabolism of microbes.

#### Mantel and partial Mantel analysis

A previous study showed that partial Mantel analysis is a robust method for evaluating the relationship among three variables^65^. This approach can control the *z*-axis and assess only the relationship between the *x*- and *y*-axes, avoiding the interaction between the *z*- and *x*-axes on the *y*-axis. In this study, Mantel analysis was employed to assess the relationships between the stability of the bacterial and fungal communities and C metabolic capacity. Stability refers primarily to the ability of the microbial community to resist translocation warming^66^. A higher similarity between the microbial communities in translocated soil compared with that in the in situ HL area indicates that the community is more resistant to translocation-related warming and that the microbial community is more stable.

#### Calculation of the microbial β-diversity

Bray-Curtis and Euclidean dissimilarity metrics were calculated to estimate the bacterial and fungal taxonomic dissimilarity (β-diversity) and environmental dissimilarity (e.g., latitude, MAT, and MAP), respectively, using the vegan package (version 2.5–6) in the R statistical program (version 4.0.2, https://www.r-project.org/)^67^. Corresponding to the 45 C metabolism ratios in soils with the same OM content, the β-diversity values of bacteria and fungi were selected to analyze the relationship between the community similarity (1-β-diversity) of bacteria and fungi and changes in microbial C metabolism.

#### Impact of the SOM content and climate change on changes in microbial communities

The distribution patterns of the bacterial and fungal communities under different SOM gradients and climatic regimes were determined through nonmetric multidimensional scaling (NMDS)^68^. To quantitatively compare the effects of the SOM gradient and climatic regimes on the bacterial and fungal community composition, three nonparametric multivariate statistical analyses were used in this study: nonparametric multivariate analysis of variance (Adonis), analysis of similarity (ANOSIM), and multiple response permutation procedure (MRPP)^69^. The linear fit between environmental dissimilarity and microbial β-diversity was analyzed using the lm function in R. A significant difference in the bacterial and fungal β-diversity among different SOM contents was evaluated by Student’s paired *t*-test using the ggpubr (version 0.4.0) package^70^. RDA was performed to analyze the relationships of bacterial and fungal communities with various environmental factors (soil geochemical attributes and climatic conditions, such as MAP and MAT). In parallel, the Monte Carlo permutation test (999 permutations) was employed to determine whether the explanation of the microbial distribution by individual factors (e.g., pH, SOC, and TN) was significant^71^.

#### Construction of the structural equation model and random forest model

A SEM was fitted to illustrate the direct or indirect effects of soil properties (e.g., pH, moisture, ammonia, and nitrate nitrogen), climate change (e.g., MAT and MAP), and bacterial and fungal β-diversity on soil C metabolic capacity^72^. Based on the Euclidean method, the changes in soil properties and climatic conditions of five translocated sites compared with those in the in situ HL site were calculated. A total of 45 ratios were obtained for each OM content. Corresponding to the 45 ratios in soils with the same OM content, the β-diversity values of bacteria and fungi were selected. The model construction process was mainly divided into three steps. In brief, these steps include the establishment of an a priori model, data normality detection, and an overall goodness-of-fit test. The prior model was constructed based on a literature review and our knowledge. For the variables that did not conform to the normal distribution, we performed logarithmic transformation. Here, we used the *χ*^2^ test (the model was assumed to exhibit a good fit if *p* > 0.05), the goodness-of-fit index (GFI; the model was assumed to show a good fit if GFI > 0.9), the root mean square error of approximation (RMSEA; the model was assumed to exhibit a good fit if RMSEA < 0.05 and *p* > 0.05)^73^ and the Bollen-Stine bootstrap test (the model was assumed to show a good fit if the bootstrap *p* > 0.10) to test the overall goodness of fit of the SEM. All SEM analyses were conducted using IBM^®^ SPSS^®^ Amos 21.0 (AMOS, IBM, USA). Additionally, the importance of the metabolic capacity of different types of C on labile and recalcitrant C was assessed by random forest models using the randomForest package (version 4.6-14) in R^74^, and the model significance and amount of interpretation were evaluated using the rfUtilities package (version 2.1–5)^75^.

### Reporting summary

Further information on research design is available in the Nature Research Reporting Summary linked to this article.

## Supplementary information

Supplementary information.

Description of Additional Supplementary Files.

Supplementary Data 1.

Reporting summary.

## Data Availability

The raw sequence data for the 16S rRNA and ITS gene amplicons were deposited in the Sequence Read Archive (SRA) at the NCBI under Accession No. PRJNA689098 and in the Genome Sequence Archive in BIG Data Center, Beijing Institute of Genomics (BIG), Chinese Academy of Sciences (http://bigd.big.ac.cn/gsa), under Accession No. CRA003750. The data supporting the findings of this study are available in Figshare at 10.6084/m9.figshare.13573430.v3^76^.

## Code availability

The figures that support the findings of this study were coded with R. The code is available at 10.6084/m9.figshare.13573430.v3^76^.
