# Supplementary material for: High stability and metabolic capacity of bacterial community promote the rapid reduction of easily decomposing carbon in soil
Source: Commun Biol. 2021 Dec 8;4:1376. doi: 10.1038/s42003-021-02907-3 (PMC8654823; doi:10.1038/s42003-021-02907-3)
Supplement: Supplementary file 1 — Supplementary information. [file 42003_2021_2907_MOESM1_ESM.pdf]

Supplementary Information: Figures S1 to S11

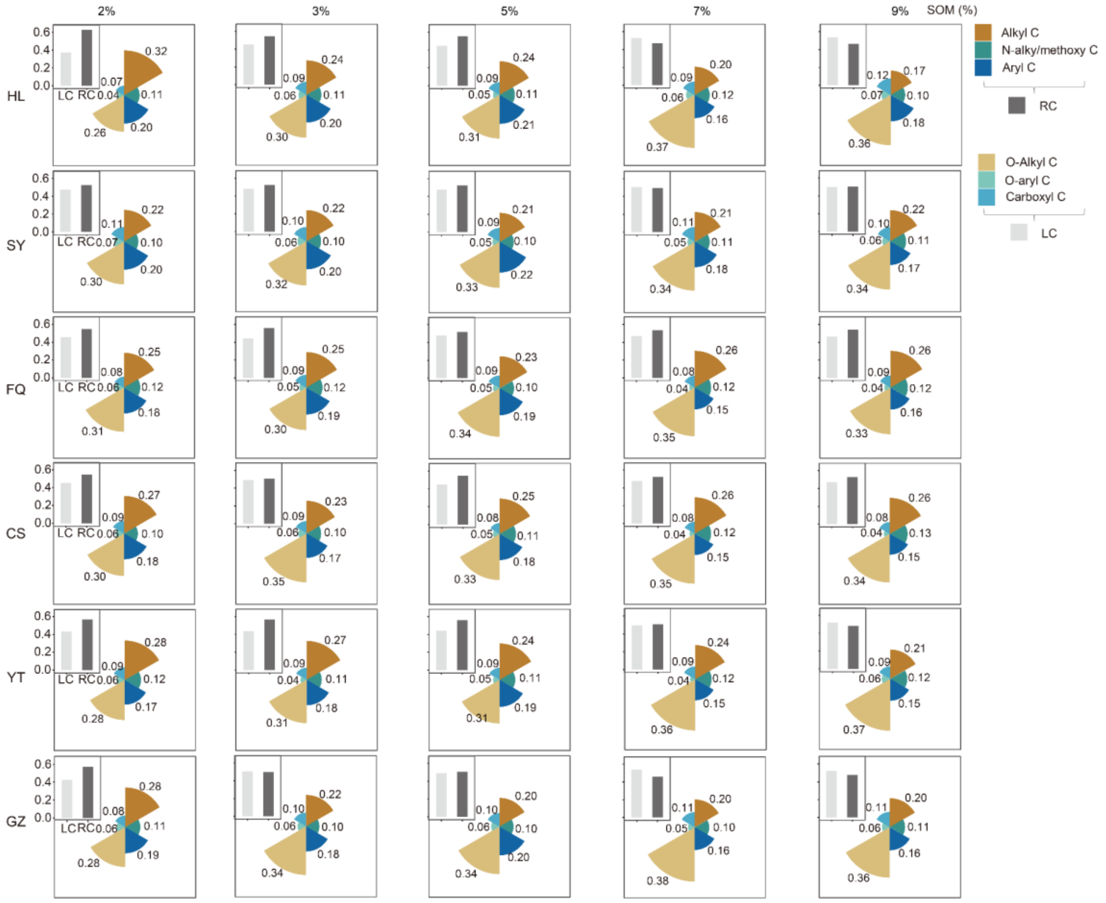

Fig. S1. The relative abundance of different C components in soils of different SOM contents from cold to warm zone. LC and RC are labile C and recalcitrant C, respectively. The value of each bar represents the proportion of that carbon components.

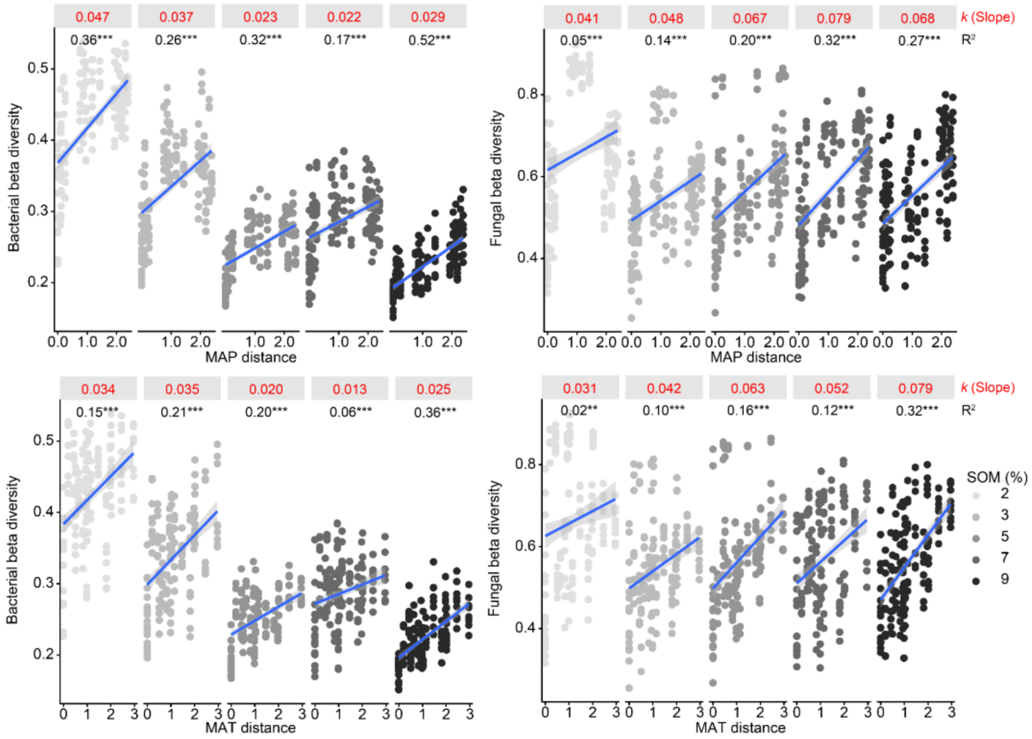

Fig. S2. Linear fitting relationships between MAP and MAT distance and bacterial and fungal β-diversity. *k* (slope) is the response coefficients of β to the distance of MAP and MAT. In this study, the response coefficient represents the rate of change in community composition. R^2 is the amount of interpretation of the model.

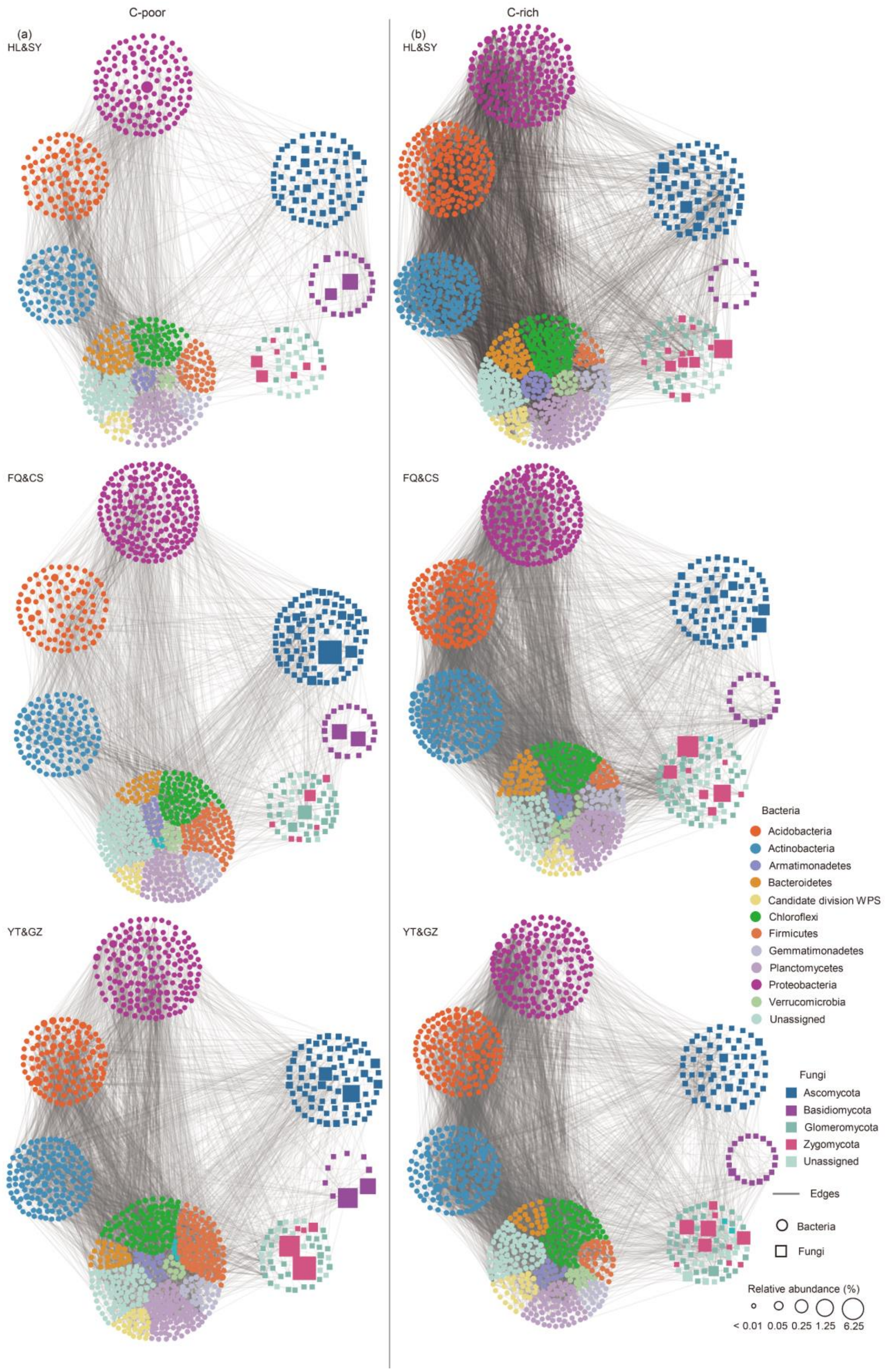

Fig. S3. Potential network interactions among soil microbial (bacterial and fungal) communities in C-poor soils (a) and C-rich soils (b). Panels HL&SY, FQ&CY and YT&GZ are the co-occurrence networks of different geoclimatic regimes from north to south. Circles are bacterial groups and quadrates are fungal groups. Node size represents the relative abundance of the species in the group. Black solid lines represent the connections among nodes.

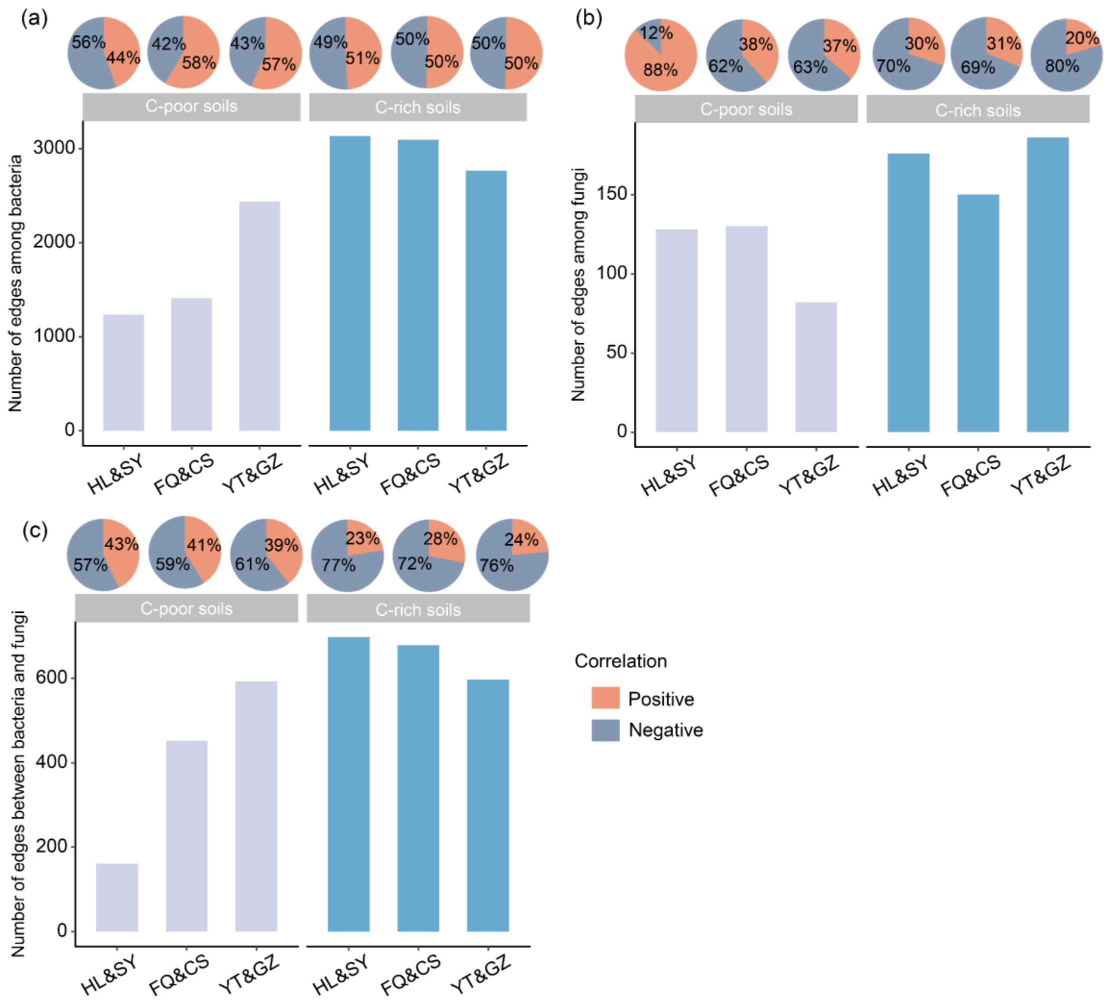

Fig. S4. Potential interactions among bacteria, fungi, and between bacteria and fungi in C-poor and C-rich soils vary with geoclimatic gradients. The histogram is the strength of the actual microbial interaction, while the pie chart is the proportion of positive and negative interactions.

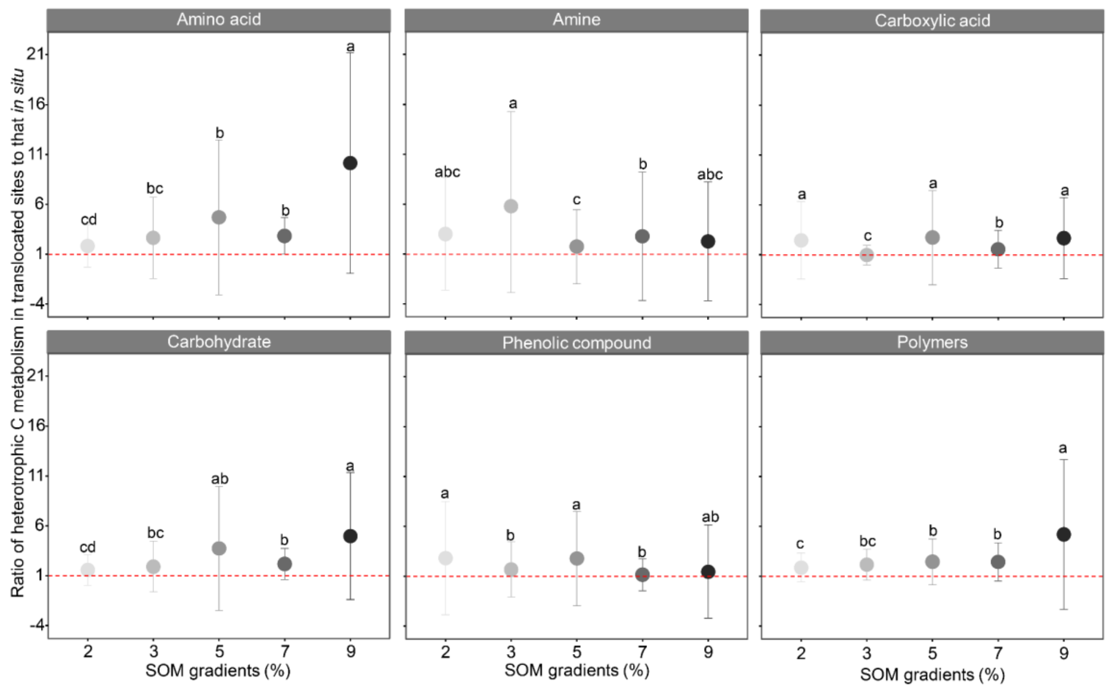

Fig. S5. Metabolic capacity of microbes to six different types of C sources in soils with different SOM gradients. Different lowercase letters indicate significant ($p < 0.05$) differences between groups, while the same lowercase letters indicate no significant ($p > 0.05$) differences. Significance is obtained by paired T test (n = 16). Error bar is the standard deviation.

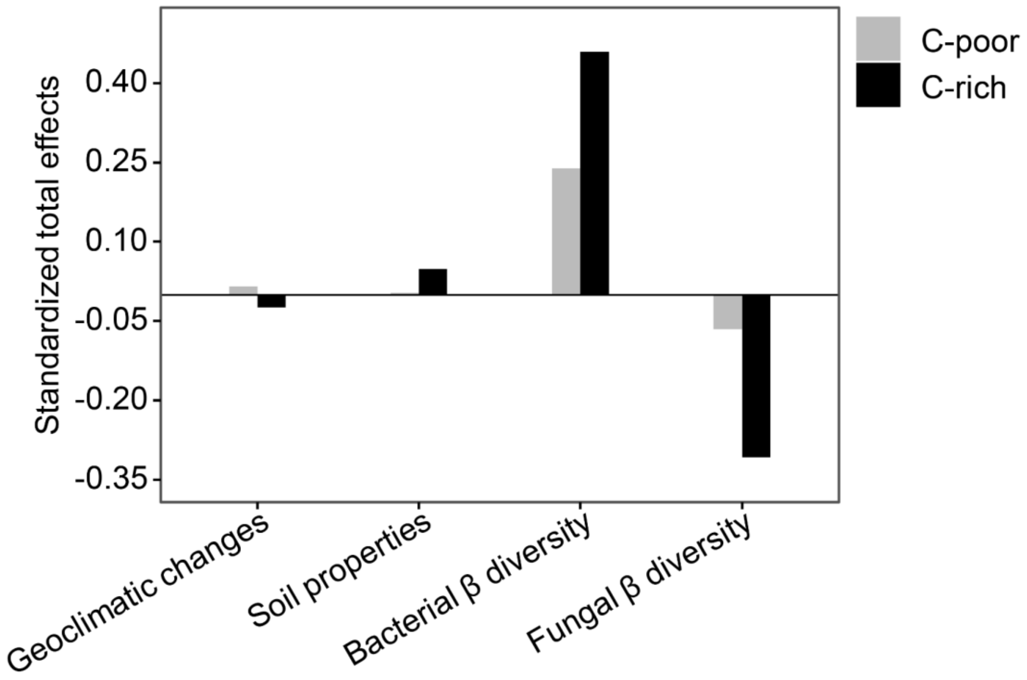

Fig. S6. Standard total effects of different factors on C metabolic profiles in C-poor and C-rich soils.

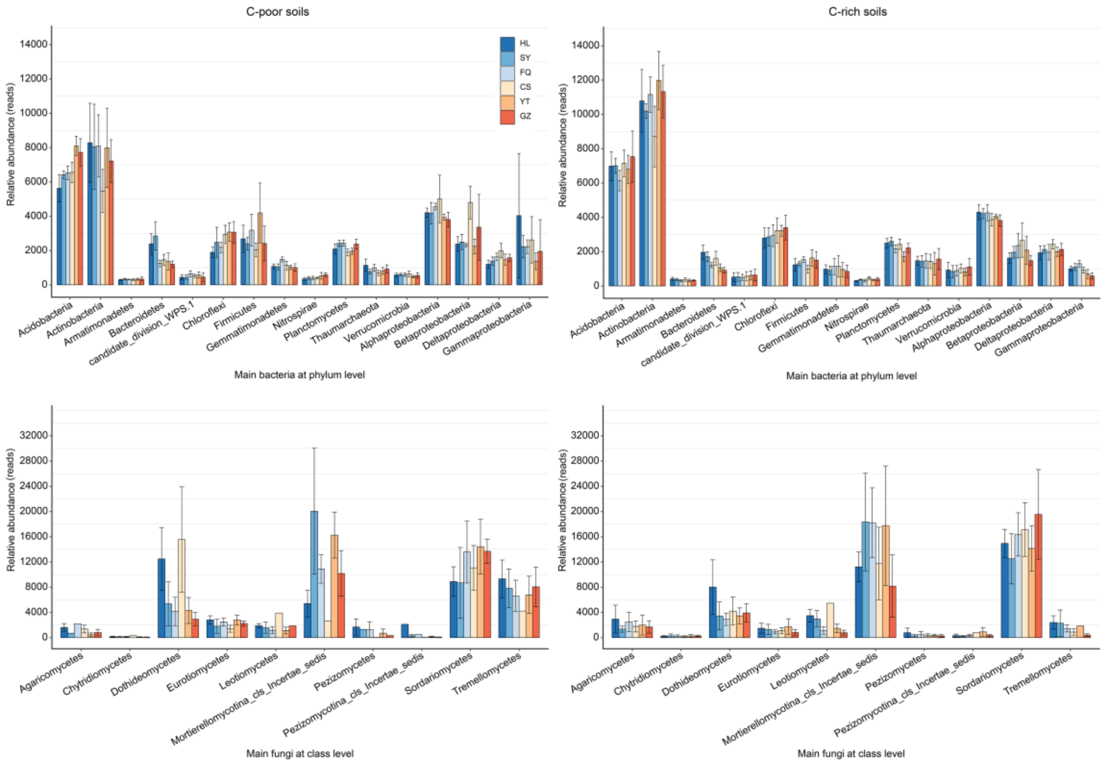

Fig. S7. Relative abundance of bacteria and fungi in C-poor and C-rich soils.

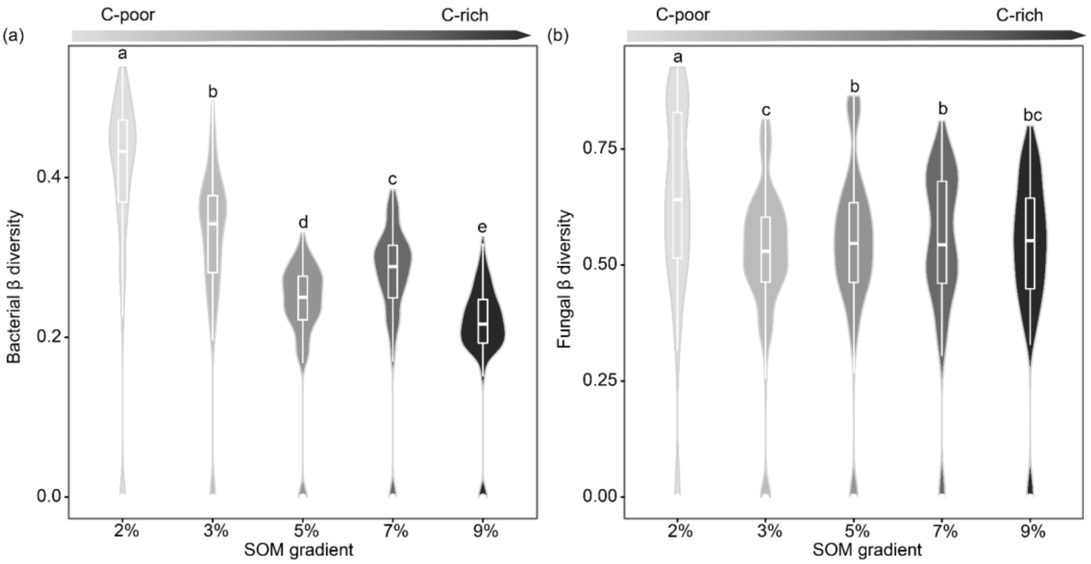

Fig. S8. Boxplot of bacterial (a) and fungal (b) β-diversity in soils with different SOM contents. Different lowercase letters represent significant differences in β-diversity among soils with different SOM contents. Significance $p < 0.001$.

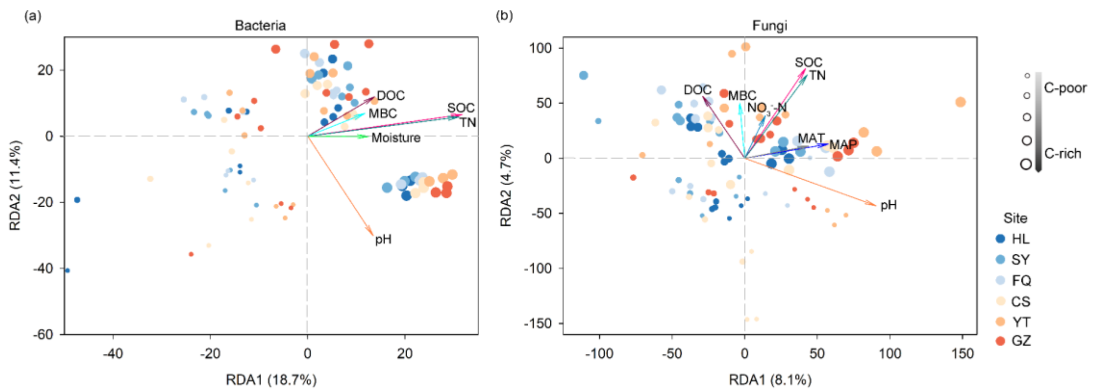

Fig. S9. Effects of different factors on the distributions of bacterial and fungal communities. Redundancy analysis (RDA) of bacterial (a) and fungal (b) communities (RDA), in which only displayed environmental factors that significantly associated with bacterial and fungal community distributions. The significance of the explanatory quantities of environmental factors for bacterial and fungal community distribution was performed by Monte Carlo permutation test.

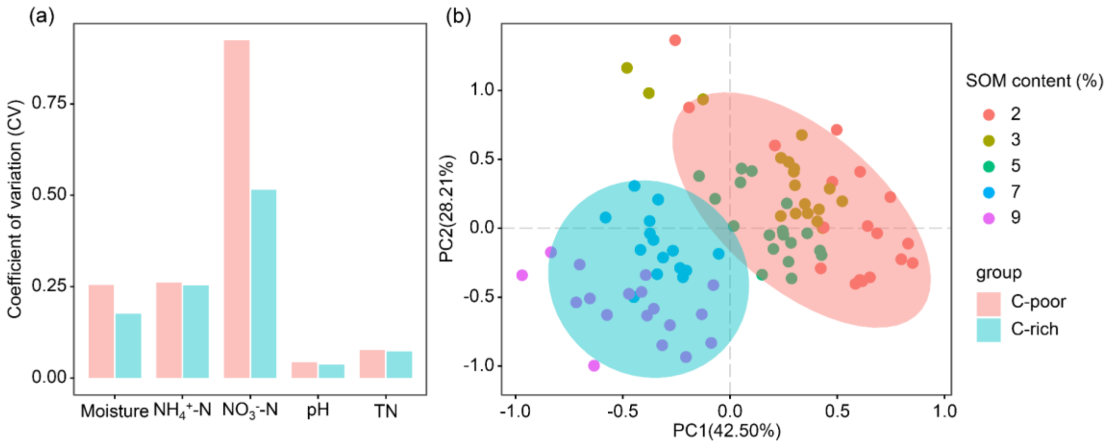

Fig. S10. Coefficient of variation of nutrient, moisture content and pH (a), and their principal component analysis (PCA) (b). The coefficient of variation is the ratio of the standard deviation to the mean in the same organic matter content.

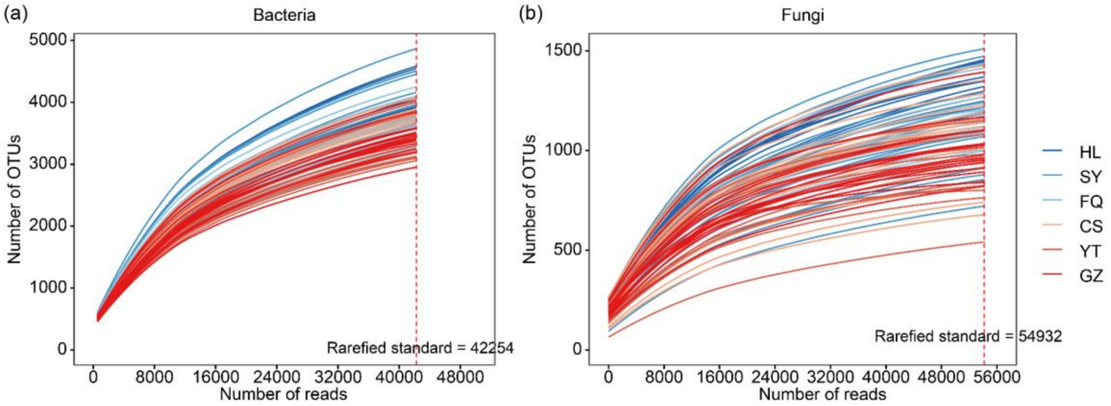

Fig. S11. Sample rarefaction curves of bacteria (a) and fungi (b).

Supplementary Information: Table S1 to S5

Table S1. Network topological parameters of different geoclimatic regimes in C-poor and C-rich soils.

	Networks	Nodes	Edges			Avg. clustering coefficient	Network density	Network diameter	Average degree
			Total	Positive (%)	Negative (%)				
C-poor soils	HL&SY	794	1763	44.98	55.02	0.138	0.005	16	4.350
	FQ&CS	1003	2233	49.84	50.16	0.128	0.004	13	4.405
	YT&GZ	1011	3324	51.05	48.95	0.167	0.006	18	6.504
C-rich soils	HL&SY	1099	4145	43.81	56.19	0.198	0.007	11	7.427
	FQ&CS	1060	4224	45.64	54.36	0.211	0.007	13	7.894
	YT&GZ	930	3737	44.23	55.77	0.185	0.009	13	7.929

Nodes represent the number of network node; edges represent the number of edge in networks, and positive (%) and negative (%) represent the proportion of the number of positively and negatively correlated edges, respectively. the Avg. clustering coefficient is the average of the clustering coefficients of all nodes in the network, it represents how well a node is connected with its neighbor; network density refers to the ratio of the number of edges actually existing in the network to the upper limit of the number of edges that can be accommodated, it reflects how densely the network is populated with edges.

Table S2. Biolog EcoPlates C source groups.

Well	C-source	Group	Well	C-source	Group
A1	Water	-	A3	D-Galactonic acid-γ-lactone	Carbohydrate
B1	Pyruvic acid methyl ester	Carboxylic acid	B3	D-Galactyronic acid	Carboxylic acid
C1	Tween 40	Polymers	C3	2-Hydroxybenzoic acid	Phenolic compound
D1	Tween 80	Polymers	D3	4-Hydroxybenzoic acid	Phenolic compound
E1	α- Cyclodextrin	Polymers	E3	γ-Hydroxybutyric acid	Carboxylic acid
F1	Glycogen	Polymers	F3	Itactonic acid	Carboxylic acid
G1	D-Cellobiose	Carbohydrate	G3	α-Ketobutyric acid	Carboxylic acid
H1	α-D-Lactose	Carbohydrate	H3	D-Malic acid	Carboxylic acid
A2	β-Methyl-D-glucoside	Carbohydrate	A4	L-Arginine	Amino acid
B2	D-Xylose	Carbohydrate	B4	L-Asparagine	Amino acid
C2	i-Erythritol	Carbohydrate	C4	L-Phenyloalanine	Amino acid
D2	D-Mannitol	Carbohydrate	D4	L-Serine	Amino acid
E2	N-Acetyl-D-glucosamine	Carbohydrate	E4	L-Threonine	Amino acid
F2	D-Glucosaminic acid	Carboxylic acid	F4	Glycyl-L-glutamin acid	Amino acid
G2	Glucose-1-phosphate	Carbohydrate	G4	Phenylethylamine	Amine
H2	D,L-α-Glycerol phosphate	Carbohydrate	H4	Putrescine	Amine

Table S3. Climatic conditions at six sites.

Site	Climatic type	Mean annual temperature (°C)	Mean annual precipitation (mm)
Hailun (HL, N 47º27′, E 126º55′)	The moderate temperate continental climate	1.5	550
Shenyang (SY, N 41º49′, E 123º33′)	The temperate monsoon climate	8.3	700
Fengqiu (FQ, N 35º03′, E 114º23′)	The warm temperate continental monsoon climate	13.5-14.5	615.1
Changshu (CS, N 31º41′, E 120º41′)	The subtropical monsoon climate	15.4	1054
Yingtan (YT, N 28º12′, E 116º55′)	The mid-subtropical humid monsoon mild climate	18.4	1750
Guangzhou (GZ, N 23º23′, E 113º27′)	The maritime subtropical monsoon climate	21.9	1736

Table S4. Soil geochemical attributes under same SOM contents.

	Site	NO_3^--N ($mg·kg^{-1}$)	NH_4^+-N ($mg·kg^{-1}$)	SOC ($g·kg^{-1}$)	DOC ($mg·kg^{-1}$)	TN ($g·kg^{-1}$)	MBC ($mg·kg^{-1}$)	Moisture (%)	pH
2%	HL	25.17±4.7 b	13.27±2.5 ab	9.68±0.2 ab	175.96±3.4 a	1.03±0.1 ab	107.91±25.6 a	13.67±2.5 a	6.07±0.1 bc
	SY	11.59±2.4 b	10.17±1.8 bc	9.30±0.4 b	128.96±2.5 b	0.95±0.1 b	59.16±29.3 a	18.67±4.1 a	6.33±0.1 b
	FQ	70.37±26.8 a	15.26±1.5 a	9.93±0.2 ab	165.03±8.4 a	1.09±0.0 a	74.83±19.1 a	17.00±2.9 a	5.81±0.1 c
	CS	2.76±0.6 b	8.49±0.7 c	9.38±0.2 b	122.95±4.3 bc	0.95±0.0 b	70.36±5.1 a	21.00±3.6 a	6.33±0.1 b
	YT	8.45±3.8 b	12.7±0.1 ab	10.04±0.3 a	110.38±8.3 c	0.98±0.0 b	79.24±40.3 a	22.33±3.3 a	6.35±0.2 b
	GZ	7.07±1.4 b	10.6±0.2 bc	9.34±0.2 b	106.56±9.1 c	0.96±0.1 b	92.06±16.3 a	21.67±3.3 a	6.66±0.1 a
3%	HL	13.28±1.9 b	16.46±0.3 b	16.21±0.4 a	196.72±10.8 ab	1.66±0.03 a	53.62±40.6 ab	17.33±1.7 b	5.60±0.1 bc
	SY	12.54±4.6 b	13.38±1.7 b	16.65±0.5 a	195.36±12.5 ab	1.47±0.1 ab	114.93±9.9 a	20.33±0.5 ab	5.57±0.0 c
	FQ	30.13±3.7 a	24.71±4.0 a	16.11±0.1 a	203.55±6.2 a	1.42±0.1 ab	63.48±47.7 ab	22.67±0.9 a	5.24±0.1 d
	CS	6.07±5.9 b	12.78±0.5 b	16.68±0.5 a	151.64±4.1 c	1.46±0.2 ab	45.64±8.4 ab	23.33±0.5 a	5.77±0.2 abc
	YT	9.12±0.2 b	11.98±0.2 b	15.81±0.1 a	144.81±20.6 c	1.35±0.1 b	31.24±9.5 b	21.67±2.1 ab	5.83±0.3 ab
	GZ	11.28±2.6 b	14.56±0.6 b	15.91±0.2 a	166.67±12.5 c	1.60±0.0 ab	48.62±18.9 ab	22.33±1.7 a	5.86±0.1 a
5%	HL	25.96±3.9 a	14.16±0.9 a	27.26±0.4 a	214.48±13.2 ab	2.47±0.1 a	68.54±12.2 ab	22.00±2.9 a	5.71±0.1 ab
	SY	9.26±3.1 c	12.92±1.6 a	26.82±0.4 ab	250.00±10.8 a	2.16±0.1 bc	161.38±25.6 a	24.00±2.2 a	5.75±0.1 ab
	FQ	23.10±4.8 ab	15.57±0.6 a	27.09±0.1 a	222.68±14.4 ab	2.31±0.2 ab	175.22±48.1 a	24.00±2.8 a	5.53±0.1 b
	CS	10.21±1.3 c	10.00±0.9 b	27.09±0.1 a	181.69±21.0 bc	2.20±0.1 abc	79.12±20.5 ab	27.33±1.7 a	5.74±0.2 ab
	YT	16.37±2.1 bc	7.91±0.8 b	26.22±0.3 bc	118.85±8.2 d	2.01±0.1 c	51.05±33.7 b	25.00±0.8 a	5.76±0.1 ab
	GZ	13.36±4.1 c	9.42±1.0 b	25.83±0.2 c	148.91±20.2 cd	2.44±0.0 ab	155.92±66.8 ab	25.33±2.5 a	5.95±0.1 a
7%	HL	21.10±4.0 b	19.16±2.3 ab	42.56±0.4 ab	426.23±22.8 a	22.82±4.0 a	164.20±47.9 a	27.33±2.4 b	5.56±0.1 a
	SY	24.79±4.4 b	16.83±1.0 bc	41.98±0.6 c	355.19±15.5 b	15.52±3.5 bc	164.51±35.7 a	28.33±2.4 b	5.62±0.0 a
	FQ	76.93±22.5 a	23.45±3.4 a	44.49±0.5 a	459.02±26.9 a	26.87±4.0 a	98.07±47.7 a	29.00±1.6 ab	5.01±0.3 b
	CS	16.32±9.8 b	12.97±1.4 cd	42.93±0.1 bc	187.16±6.3 c	6.26±3.6 bc	81.67±14.1 a	35.67±2.5 a	5.55±0.1 a
	YT	23.24±5.1 b	10.87±0.7 d	40.67±0.6 d	133.88±8.5 d	8.53±3.3 c	83.03±27.6 a	31.33±2.4 ab	5.70±0.0 a
	GZ	24.14±4.4 b	13.74±1.3 ad	40.35±0.1 d	177.60±8.53 cd	8.53±3.7 ab	102.28±29.6 a	31.00±0.8 ab	5.63±0.0 a
9%	HL	24.20±0.4 bc	19.15±3.7 a	53.61±0.3 a	277.3±27.9 ab	4.67±0.2 ab	100.96±36.4 ab	23.67±1.9 b	6.91±0.1 a
	SY	22.61±8.8 bc	16.45±3.2 ab	53.54±0.6 b	323.77±20.5 a	4.55±0.1 ab	134.61±66.2 a	26.00±5.1 ab	6.55±0.1 b
	FQ	52.35±10.1 a	27.37±13.2 a	54.87±0.6 bc	256.83±31.3 ab	5.09±0.1 a	110.29±25.7 ab	29.33±2.6 ab	6.31±0.1 c
	CS	29.48±2.2 b	17.19±1.4 ab	54.90±0.7 bc	301.91±34.9 ab	4.57±0.1 ab	176.52±28.9 a	31.67±1.2 ab	6.37±0.1 bc
	YT	30.91±4.0 b	12.08±1.6 b	53.39±0.6 b	176.23±14.8 c	4.48±0.3 b	99.85±12.2 ab	34.67±4.1 a	6.31±0.1 c
	GZ	15.36±1.1 c	13.23±3.1 ab	51.10±0.6 c	239.07±38.1 bc	4.60±0.23 ab	33.86±4.8 b	29.67±0.9 ab	6.50±0.1 bc

The values in the table are mean ± standard deviation. Significance between different factors in soils with the same organic matter content was tested using the TukeyHSD method. Different lowercase letters indicate significant differences

Table S5. Climatic conditions at six sites.

Site	Climatic type	Mean annual temperature (°C)	Mean annual precipitation (mm)
Hailun (HL, N 47º27′, E 126º55′)	The moderate temperate continental climate	1.5	550
Shenyang (SY, N 41º49′, E 123º33′)	The temperate monsoon climate	8.3	700
Fengqiu (FQ, N 35º03′, E 114º23′)	The warm temperate continental monsoon climate	13.5-14.5	615.1
Changshu (CS, N 31º41′, E 120º41′)	The subtropical monsoon climate	15.4	1054
Yingtan (YT, N 28º12′, E 116º55′)	The mid-subtropical humid monsoon mild climate	18.4	1750
Guangzhou (GZ, N 23º23′, E 113º27′)	The maritime subtropical monsoon climate	21.9	1736
