# Supplementary material for: High stability and metabolic capacity of bacterial community promote the rapid reduction of easily decomposing carbon in soil
Source: Commun Biol. 2021 Dec 8;4:1376. doi: 10.1038/s42003-021-02907-3 (PMC8654823; doi:10.1038/s42003-021-02907-3)
Supplement: Supplementary file 2 — Description of Additional Supplementary Files. [file 42003_2021_2907_MOESM2_ESM.pdf]

Description of Additional Supplementary Files

File name: Supplementary Data 1.
Description: Supplementary data of soil physiochemical properties, climatic conditions (Sheet 1), and solid-state NMR spectroscopy (Sheet 2).
