# Supplementary material for: High stability and metabolic capacity of bacterial community promote the rapid reduction of easily decomposing carbon in soil
Source: Commun Biol. 2021 Dec 8;4:1376. doi: 10.1038/s42003-021-02907-3 (PMC8654823; doi:10.1038/s42003-021-02907-3)
Supplement: Supplementary file 4 — Reporting summary. [file 42003_2021_2907_MOESM4_ESM.pdf]

nature research

Corresponding author(s): Yuting Liang

Last updated by author(s): Mar 31, 2021

Reporting Summary

Nature Research wishes to improve the reproducibility of the work that we publish. This form provides structure for consistency and transparency in reporting. For further information on Nature Research policies, see our Editorial Policies and the Editorial Policy Checklist.

Statistics

For all statistical analyses, confirm that the following items are present in the figure legend, table legend, main text, or Methods section.

n/a	Confirmed	
☐	☒	The exact sample size (*n*) for each experimental group/condition, given as a discrete number and unit of measurement
☐	☒	A statement on whether measurements were taken from distinct samples or whether the same sample was measured repeatedly
☐	☒	The statistical test(s) used AND whether they are one- or two-sided<br>*Only common tests should be described solely by name; describe more complex techniques in the Methods section.*
☐	☒	A description of all covariates tested
☐	☒	A description of any assumptions or corrections, such as tests of normality and adjustment for multiple comparisons
☐	☒	A full description of the statistical parameters including central tendency (e.g. means) or other basic estimates (e.g. regression coefficient) AND variation (e.g. standard deviation) or associated estimates of uncertainty (e.g. confidence intervals)
☒	☐	For null hypothesis testing, the test statistic (e.g. *F*, *t*, *r*) with confidence intervals, effect sizes, degrees of freedom and *P* value noted<br>*Give P values as exact values whenever suitable.*
☒	☐	For Bayesian analysis, information on the choice of priors and Markov chain Monte Carlo settings
☐	☒	For hierarchical and complex designs, identification of the appropriate level for tests and full reporting of outcomes
☐	☒	Estimates of effect sizes (e.g. Cohen's *d*, Pearson's *r*), indicating how they were calculated

Our web collection on statistics for biologists contains articles on many of the points above.

Software and code

Policy information about availability of computer code

Data collection	No software used for data collection
Data analysis	FLASH, v1.2.11, QIIME2, SILVA, UNITE and RDP Classifier were used to process the sequencing data; R (v4.0.2) was used for statistical analyses; Cytoscape (version 3.8.1) was used for network visualization;SEM analyses were conducted using IBM® SPSS® Amos 21.0 (AMOS IBM USA).

For manuscripts utilizing custom algorithms or software that are central to the research but not yet described in published literature, software must be made available to editors and reviewers. We strongly encourage code deposition in a community repository (e.g. GitHub). See the Nature Research guidelines for submitting code & software for further information.

Data

Policy information about availability of data

All manuscripts must include a data availability statement. This statement should provide the following information, where applicable:

- Accession codes, unique identifiers, or web links for publicly available datasets
- A list of figures that have associated raw data
- A description of any restrictions on data availability

Raw sequence data for 16S rRNA and ITS gene amplicons were deposited in the Sequence Read Archive (SRA) at the NCBI under accession number No. PRJNA689098, and in the Genome Sequence Archive in BIG Data Center, Beijing Institute of Genomics (BIG), Chinese Academy of Sciences (http://bigd.big.ac.cn/gsa), under accession number No. CRA003750, respectively. The data that support the findings of this study are available in Figshare at https://doi.org/10.6084/m9.figshare.13573430.v1

Field-specific reporting

Please select the one below that is the best fit for your research. If you are not sure, read the appropriate sections before making your selection.

☐ Life sciences ☐ Behavioural & social sciences ☒ Ecological, evolutionary & environmental sciences

For a reference copy of the document with all sections, see nature.com/documents/nr-reporting-summary-flat.pdf

Ecological, evolutionary & environmental sciences study design

All studies must disclose on these points even when the disclosure is negative.

Study description	In this study, agricultural soils with five SOM gradients were collected from Northeast China in 2015. SOM contents were 2%, 3%, 5%, 7% and 9% (equivalent to 10, 18, 28, 36, and 56 g C kg-1 soil-1) and all soils are classified as Mollisol according to FAO classification. Here, we designed a unique latitudinal soil transplantation experiment to investigate the relationship between the changes in bacterial and fungal community composition and the responses of soil C molecular structure to abrupt climate change.
Research sample	The research sample was the soil sample in situ and transplanted. The 16S rRNA and ITS sequence data were obtained from the soil sample. Soil microbial carbon metabolic activities were also measured.
Sampling strategy	Soil samples were collected from the six ecological research stations: Hailun Agricultural Ecological Experimental Station (HL, N 47° 27′, E 126°55′) in Heilongjiang Province, Shenyang Agriculture Ecological Experimental Station (SY, N 41°49′, E 123°33′) in Liaoning Province, Fengqiu Agricultural Ecological Experimental Station (FQ, N 35°03′, E 114°23′) in Henan Province, Changshu Agricultural Ecological Experimental Station (CS, N 31°41′, E 120°41′) in Jiangsu Province, Yingtan Red Soil Ecological Experiment Station (YT, N 28° 12′, E 116°55′) in Jiangxi Province and Guangzhou National Agricultural Science and Technology Park (GZ, N 23°23′, E 113°27′) in Guangdong Province, respectively. The mean annual temperature (MAT) and mean annual precipitation (MAP) at the six ecological research stations are ranged from 1.5 °C to 21.9 °C and from 550 mm to 1750 mm from north to south, respectively. Detail of their climatic conditions (e.g., climatic types) are shown in Table S3. All the tubes were taken from each station after one year.
Data collection	Solid-state 13C nuclear magnetic resonance (NMR) spectroscopy analysis was used to determine the molecular structure of SOC. All geochemical attributes were measured by the coauthors. Soil microbial C metabolic profiles were measured with BIOLOG 96-well Eco-Microplates (Biolog Inc., USA). Taxonomic profiling of the soil bacterial and fungal communities was performed using an Illumina® HiSeq Benchtop Sequencer.
Timing and spatial scale	The sampling was carried out in October to November 2015. Soil samples were taken from the six ecological research stations from HL, N 47°27′, E 126°55′ to GZ, N 23°23′, E 113°27′.
Data exclusions	No data were excluded.
Reproducibility	No laboratory manipulation experiments of biological processes were conducted - rather, and as stated above, our analyses are based on one-time survey conducted on the six ecological research stations.
Randomization	No experiments per se were conducted, there was thus no experimental group allocation. There was no further group partitioning of data beyond the natural groupings associated with geography.
Blinding	Blinding was not relevant to our study, because all available data were used (our study did not perform an experiment).

Did the study involve field work? ☒ Yes ☐ No

Field work, collection and transport

Field conditions	The mean annual temperature (MAT) and mean annual precipitation (MAP) at the six ecological research stations are ranged from 1.5 °C to 21.9 °C and from 550 mm to 1750 mm from north to south, respectively.
Location	Hailun Agricultural Ecological Experimental Station (HL, N 47°27′, E 126°55′) in Heilongjiang Province, Shenyang Agriculture Ecological Experimental Station (SY, N 41°49′, E 123°33′) in Liaoning Province, Fengqiu Agricultural Ecological Experimental Station (FQ, N 35° 03′, E 114°23′) in Henan Province, Changshu Agricultural Ecological Experimental Station (CS, N 31°41′, E 120°41′) in Jiangsu Province, Yingtan Red Soil Ecological Experiment Station (YT, N 28°12′, E 116°55′) in Jiangxi Province and Guangzhou National Agricultural Science and Technology Park (GZ, N 23°23′, E 113°27′) in Guangdong Province, respectively. Soils were taken from the PVC pipes translocated.
Access & import/export	All sampling sites are long-term scientific research experimental stations. A consensus was reached before the experiment was set up.
Disturbance	Once the experiment was set up, in order to avoid the impact of plants, the weeds that may grow in each PVC pipe were manually removed every 2-3 weeks.

Reporting for specific materials, systems and methods

We require information from authors about some types of materials, experimental systems and methods used in many studies. Here, indicate whether each material, system or method listed is relevant to your study. If you are not sure if a list item applies to your research, read the appropriate section before selecting a response.

Materials & experimental systems

n/a	Involved in the study
☒	☐ Antibodies
☒	☐ Eukaryotic cell lines
☒	☐ Palaeontology and archaeology
☒	☐ Animals and other organisms
☒	☐ Human research participants
☒	☐ Clinical data
☒	☐ Dual use research of concern

Methods

n/a	Involved in the study
☒	☐ ChIP-seq
☒	☐ Flow cytometry
☒	☐ MRI-based neuroimaging
